# Femoral neck fracture after femoral head necrosis: a case report and review of the literature

**DOI:** 10.1186/s12891-023-06992-9

**Published:** 2023-10-31

**Authors:** Wenjie Xia, Aiqi Zhang, Binsong Qiu, Yuan Chen, Mingxiang Kong

**Affiliations:** 1 General Surgery, Cancer Center, Department of Breast Surgery, Zhejiang Provincial People’s Hospital, Affiliated People’s Hospital, Hangzhou, Zhejiang China; 2 The second Clinical Medical College of Zhejiang Chinese Medical University, Hangzhou, Zhejiang China; 3 Center for Rehabilitation Medicine, Department of Orthopedics, Zhejiang Provincial People’s Hospital, Affiliated People’s Hospital, Hangzhou Medical College, Hangzhou, Zhejiang China; 4 Cancer Center, Department of Pathology, Zhejiang Provincial People’s Hospital, Affiliated People’s Hospital, Hangzhou Medical College, Hangzhou, Zhejiang China

**Keywords:** Femoral neck fracture, Rapidly destructive hip disease, Femoral head osteonecrosis, Osteoporosis, Total hip replacement

## Abstract

**Introduction:**

Pathological fractures of the femoral neck caused by necrosis of the femoral head are extremely rare. Here, we report a rare case of bilateral femoral head osteonecrosis extending to the femoral neck, with bilateral pathological fractures of the femoral neck occurring within a short period of time.

**Case report:**

A 65-year-old male with a 25-year history of daily consumption of 750 ml of liquor, presented with right hip pain after labor for 1 month. He subsequently sustained a right femoral neck fracture without trauma and underwent a right total hip arthroplasty. Two months later, he suffered a non-traumatic left femoral neck fracture and underwent a left total hip arthroplasty. Histopathological examination revealed osteonecrosis of the femoral head and neck, along with the presence of osteoclasts and granulomatous inflammation. Bone mineral density testing also showed osteoporosis. The bilateral femoral neck fractures were ruled out to be caused by any other pathological factors.

**Discussion:**

This is the first report of pathological fractures of the bilateral femoral neck caused by femoral head necrosis. During the literature review process, we found that this case conforms to the histological characteristics of rapidly destructive hip disease and analyzed the etiology of femoral head necrosis and the pathogenesis of femoral neck fractures.

## Introduction

Necrosis of the femoral head is a common cause of hip pain. It can be categorized into traumatic and non-traumatic causes [1, 2]. Traumatic necrosis is mainly caused by femoral neck fractures or hip dislocations, which can damage the local blood vessels of the femoral head, leading to bone necrosis [3]. The pathogenesis of non-traumatic femoral head necrosis is not yet clear, but it is generally believed to be related to the use of large amounts of oral corticosteroids and heavy alcohol consumption [4]. Necrosis of the femoral head is a lengthy process and can lead to subchondral fractures, resulting in the appearance of the crescent sign [3]. Here, we report a rare case of a 65-year-old male patient who had been drinking heavily for years and developed avascular necrosis of the femoral head and neck in a short period of time. However, there was no obvious subchondral fracture or crescent sign, and he subsequently suffered from bilateral femoral neck fractures without any apparent traumatic factors. Previous studies have not reported cases of bilateral non-traumatic avascular necrosis of the femoral head with femoral neck fractures. This study aims to provide clinical practitioners with a reference for diagnosing the etiology of this rare condition by reporting such a case.

## Case report

**Fig. 1:**
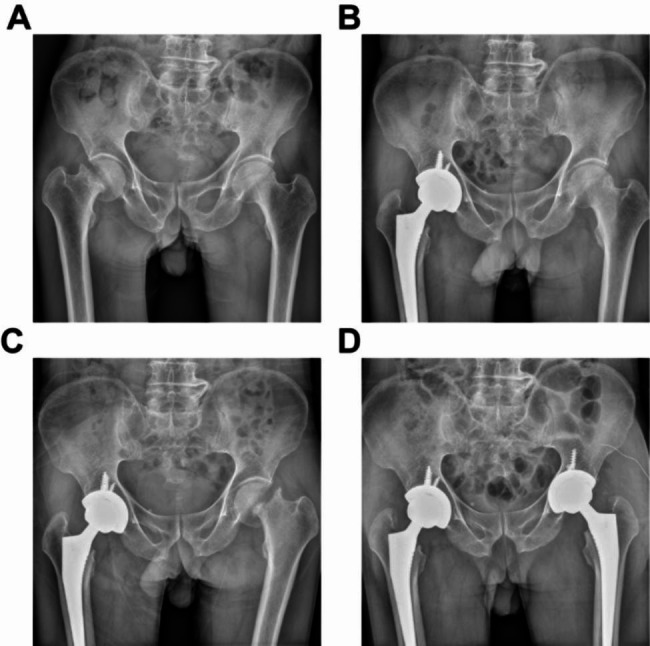
Anteroposterior radiographs of the patient’s hip joint before and after surgery

**Fig. 2:**
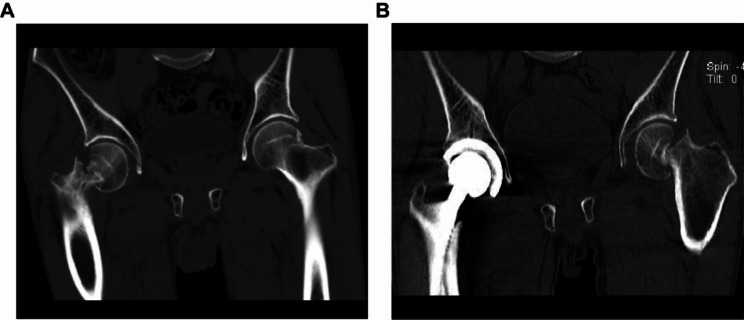
Patient hip CT

**Fig. 3:**
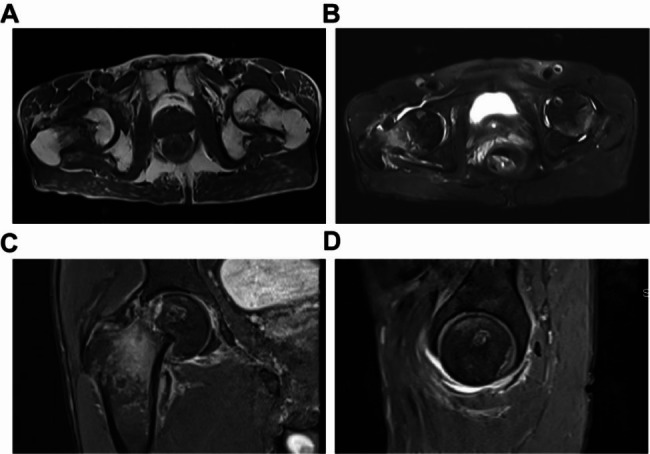
Hip joint MRI of a patient with first-time femoral neck fracture. (**A**) The T1-weighted image revealed low signal intensity in the right femoral neck and linear low signal intensity in the left femoral neck, which may be indicative of necrosis. (**B**) The T2-weighted image reveals the presence of subchondral edema in the right femoral head, high signal intensity in the femoral neck indicating femoral neck fracture, and local high signal intensity in the left femoral head without any evidence of trauma. (**C**-**D**) T2-weighted images revealed subchondral edema in both femoral heads, indicating osteonecrosis. There was also edema in the femoral neck on the right side, which was caused by a fracture of the femoral neck

**Fig. 4:**
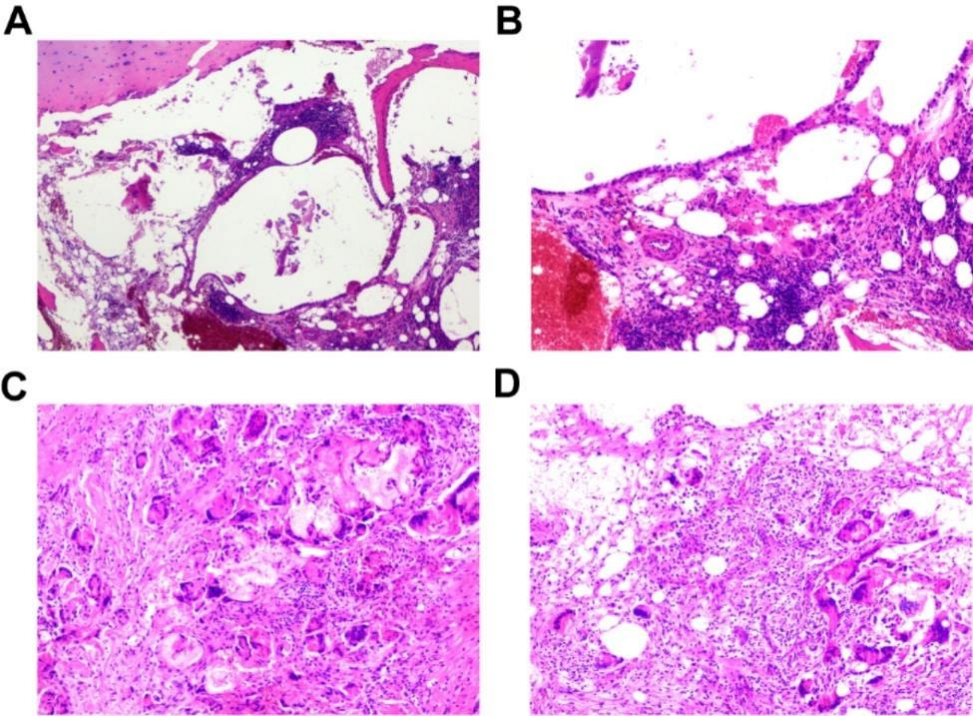
Pathology of the patient’s left femoral head (**A**-**B**) and femoral neck (**C**-**D**)(hematoxylin-eosin staining) (**A**) There is necrosis of the subcapital cartilage of the femoral head, disordered trabecular structure in part of the bone, and necrosis of the bone marrow. (**B**) An enlarged image on the left shows a small number of osteoclast giant cells and a large number of inflammatory cells. (**C** and **D**) There is a disordered arrangement of bone trabeculae, formation of dead bone, an increased number of macrophages, granuloma formation, and the presence of osteoclastic giant cells associated with bone fragments

The patient was informed that their data would be made public and they agreed. The patient was a 65-year-old male with a height of 160 cm, weight of 45 kg, and a body mass index (BMI) of 17.5 kg/m^2^. He presented with dull pain in the right hip during labor, with tenderness in the anterior aspect of the hip joint. Two weeks after onset, the patient’s pain intensified, and an X-ray of the right hip revealed a subcapital femoral fracture (Fig. 1). A computed tomography (CT) scan of the right hip also showed a subcapital femoral fracture (Fig. 2). The patient reported a 25-year history of daily alcohol consumption, with three glasses of white wine (approximately 250 mL) per day, and a 20-year history of smoking, with approximately 5 cigarettes per day. To rule out the possibility of femoral head necrosis, the patient underwent a magnetic resonance imaging (MRI) of the hip. The MRI examination revealed the presence of subchondral edema in both femoral heads, indicating arthritis and necrosis (Fig. 3). A right femoral neck fracture and abnormal signal in the left femoral neck were also noted. The patient underwent right total hip replacement surgery. Follow-up X-rays of the right hip after the surgery were obtained (Fig. 1). Histopathological examination of the femoral head tissue revealed necrosis and inflammatory exudation on the surface of the femoral head, synovial tissue hyperplasia, chronic inflammation, and hemosiderin deposition, with dead bone formation in some areas and necrosis and hemorrhage in the bone marrow tissue (Fig. 4). This indicated that the patient had already experienced necrosis in some areas of the femoral head. We advised the patient to quit smoking and drinking. Regular outpatient follow-up was recommended after the patient was discharged. However, the patient did not quit smoking or drinking.

The patient experienced left hip pain and discomfort after lifting heavy objects two months after discharge. One week later, the pain worsened and was unbearable, with significant aggravation during walking and an inability to straighten the left leg when lying flat, causing severe pain that prevented walking. Left hip X-rays showed a subcapital femoral fracture (Fig. 1), and a left hip CT scan confirmed the diagnosis of subcapital femoral fracture (Fig. 2). The patient underwent left total hip arthroplasty. Postoperative pathological biopsies were performed on the femoral head and neck tissues, and the histopathological results indicated the presence of necrotic lesions in both the femoral head and neck. In addition, a large number of osteoclasts and granulomas were found in the femoral neck (Fig. 4). Postoperative left hip X-ray (Fig. 1). To investigate the cause of bilateral femoral neck fractures, the patient underwent dual-energy X-ray absorptiometry, revealing a T-score of -2.9 for the left femoral neck and − 3.5 for the lumbar spine, indicating osteoporosis. The patient’s parathyroid hormone level was 13.5 pg/mL (normal range 15–65), while β-C-telopeptide of type I collagen β (CTX-Iβ) was 766.5 pg/mL (normal range <704), and N-terminal propeptide of type I procollagen (PINP), bone calcium, and vitamin D levels were normal. Thyroid function was normal, and ultrasound of the parathyroid and thyroid regions showed no positive findings. The patient has no history of non-steroidal anti-inflammatory drugs or corticosteroid use. In addition, postoperative pathological examination also ruled out factors such as tumors and infections.

## Discussion

As far as we know, this is the first reported case of pathological fracture of the bilateral femoral neck following femoral head necrosis. Only one rare case of extensive necrosis of the femoral head extending to the femoral neck in a short period of time has been reported in the literature, but there was no secondary femoral neck fracture [5]. Two cases of epiphyseal displacement similar to femoral neck fracture have been reported. Shoji Baba et al. [6]reported a case of a long-term steroid user with necrosis of the femoral head, which resulted in epiphyseal displacement of the femoral head shaft. The authors suggest that stress concentration in the lateral portion of the femoral epiphyseal leads to abnormal shear stress in the epiphyseal scar, resulting in a femoral epiphyseal slip fracture. Takanori Miura et al. [7]reported an 86-year-old female with osteoporosis who developed epiphyseal displacement of the femoral head shaft following necrosis of the femoral head. The author proposed that such cases represent a rapidly destructive hip disease (RDHD). Considering the characteristics of the disease reported in previous literature and in our case, we hypothesize that our patient had RDHD and that abnormal shear stress acting on the weakened area of the femoral neck due to osteoporosis led to bilateral femoral neck fractures.

Rapidly destructive hip disease (RDHD) is a progressive hip joint disease that results in rapid destruction. The natural history of RDHD includes rapid and progressive bone destruction, which typically occurs within 6–12 months of the onset of symptoms, even in the absence of significant pre-existing anatomical abnormalities or mild bone and joint changes [8]. The disease is often accompanied by osteoporosis [9, 10]. Lequesne et al. [11]first proposed the concept of RDHD in 1970 and defined it as a joint space reduction of 50% or more or a loss of articular cartilage greater than 2 mm within one year. The exact nature of this disease is not fully understood, with some reports suggesting it is a type of femoral head necrosis [12, 13], while others believe it is a type of osteoarthritis [14, 15]. Yamamoto et al. found granulomatous lesions in the bone marrow of RDHD and considered the presence of bone fragments and articular cartilage debris to be a characteristic feature of RDHD [16]. A histological and morphological study of 15 RDHD patients found a large number of granulomas in the bone marrow and synovium, with abnormal activation of osteoclasts in synovial fluid being a major cause of femoral head and acetabular bone destruction [17]. However, the cause of abnormal osteoclast activation remains unclear. Yamakawa et al. [18] studied RDHD femoral head sections and found that the middle surface of the femoral head was significantly vascularized and contained a large number of osteoclasts, which may be related to the development of RDHD. In addition, Soskolne et al. found that osteoclasts form synapses that are directly connected to vascular endothelial cells [19]. Therefore, the accumulation and abnormal activation of osteoclasts in the bone marrow and synovium of RDHD patients may be one of the important mechanisms of RDHD pathogenesis.

Our patient presented with non-traumatic femoral neck fracture on the right side after one month of hip pain. Two months after receiving total hip replacement therapy, the patient developed non-traumatic femoral neck fracture on the contralateral side, and the disease progressed rapidly. The main MRI features of RDHD include extensive bone marrow edema in the femoral head and neck, flattening of the femoral head, and cystic-like subchondral defects [14]. Our study demonstrated the presence of irregular low signal lines on the left femoral head and femoral neck in the T1-weighted phase of MRI, indicating localized osteonecrosis. The T2-weighted phase revealed a bone marrow edema signal extending from the femoral head to the femoral neck, which is consistent with the MRI features of RDHD. Histopathological analysis of the femoral head and neck revealed bone necrosis, which was formed by osteoclasts and granulation tissue, and elevated CTX-Iβ suggested that osteoclasts were abnormally activated. In addition, our case had a long history of heavy alcohol consumption. Previous studies have shown that excessive alcohol consumption also induces the expression of genes related to osteoclast differentiation [20]. Therefore, excessive alcohol consumption may be one of the reasons for osteoclast activation in this case. Therefore, based on the patient’s history, imaging findings, and histological features, we believe that our patient may have a more specific form of RDHD. In our case, flattening of the femoral head and subchondral defects were not found, which may be due to bilateral femoral neck fractures that occurred early in the disease process and disrupted the natural course of the disease.

The bone trabeculae within the necrotic lesion showed fracture and disorganized arrangement, with local vascular and fibrous tissue proliferation leading to the formation of scar tissue and decreased stress resistance. Our case also exhibited osteoporosis in the left femoral neck (bone mineral density T-score − 2.9), likely resulting from long-term alcohol consumption, smoking, and advanced age, which are known risk factors for the condition [21, 22]. PINP and CTX-Iβ are commonly used clinical markers of bone formation and resorption, respectively [23]. We found that the patient had elevated levels of CTX-Iβ at 766.5 pg/ml (normal range < 704), as well as a low level of parathyroid hormone (PTH) at 13.5 pg/ml (normal range 15–65 pg/ml). PTH exerts its effects by binding to PTH receptors and directly stimulating osteoblast differentiation and proliferation [24]. Therefore, the low level of PTH may also contribute to the patient’s osteoporosis. In addition, the presence of numerous granulomas and osteoclasts in the femoral neck suggests strong bone resorption activity in this area, which may have contributed to the pathological fracture. Abnormal shear stress can be generated when stress is concentrated along the weak area of the femoral neck located on the outer side of the femoral trochanter [6], which may have also contributed to the fracture in this case. Therefore, the combination of femoral head necrosis and osteoporosis, as well as the presence of abnormal shear stress, may be the underlying causes of the pathological fracture in this patient, and the mechanism of bilateral fractures may be similar. In this case, the patient underwent total hip arthroplasty and achieved favorable clinical outcomes. Furthermore, recent studies have indicated that ultra-short non-cemented total hip arthroplasty can be used for the treatment of femoral head necrosis, and the mid-term clinical and radiological outcomes are satisfactory [25].

This study has some limitations. Firstly, we did not analyze markers such as prostaglandins and proteolytic enzymes in the patients’ synovial fluid, which have been reported to be elevated and can aid in distinguishing primary hip joint diseases [26]. Additionally, due to the limited number of cases (only one patient), we were unable to comprehensively discuss all the potential causes of RDHD reported in the literature.

In conclusion, we report a case of bilateral femoral head necrosis resulting in pathologic fracture of the femoral neck, which may represent a rapidly progressive bone and joint disease. Our case highlights the importance of recognizing the potential for femoral neck fracture in patients with femoral head necrosis and osteoporosis, which may progress to RDHD. As the aging population grows, the number of these patients is likely to increase. Early diagnosis of femoral neck fracture in these patients allows for timely surgical intervention with total hip arthroplasty. Therefore, a detailed evaluation, including MRI and careful follow-up, is necessary in elderly patients with hip pain.

## Data Availability

The datasets used during the current study are available from the corresponding author on reasonable request.

## Abbreviations

CT: Computed tomography
MRI: Magnetic resonance imaging
CTX-Iβ: β-C-telopeptide of type I collagen β
PINP: N-terminal propeptide of type I procollagen
RDHD: Rapidly destructive hip disease
PTH: Parathyroid hormone
